# ‘Take my kidneys but not my corneas’—Selective preferences as a hidden problem for ‘opt‐out’ organ donation policy

**DOI:** 10.1111/bioe.13052

**Published:** 2022-05-27

**Authors:** Nicola Jane Williams, Neil C. Manson

**Affiliations:** ^1^ Department of Politics, Philosophy and Religion Lancaster University Lancaster UK

**Keywords:** deemed consent, familial consent to organ donation, opt‐out policy defaults, post‐mortem organ donation, presumed consent, selective organ donation preferences

## Abstract

With aims to both increase organ supply *and* better reflect individual donation preferences, many nations worldwide have shifted from ‘opt‐in’ to ‘opt‐out’ systems for post‐mortem organ donation (PMOD). In such countries, while a prospective donor's willingness to donate their organs/tissues for PMOD was previously ascertained—*at least partially*—by their *having recorded positive donation preferences* on an official register prior to death, this willingness is now presumed or inferred—*at least partially*—from their *not having recorded an objection to PMOD*—on an official organ donation register. Using evidence regarding the presence and prevalence of selective donation preferences, and via exploration of *how appeals to donation preferences are used to both motivate and legitimate* shifts to opt‐out frameworks, this paper draws attention to a set of previously unexplored problems for opt‐out organ donation arising in contexts where: (a) individuals demonstrate selective post‐mortem organ/tissue donation preferences, (b) legislation provides prospective donors with the opportunity to *selectively permit*/*refuse* the donation of certain organs/tissues in line with these preferences. While selective preferences pose few problems for opt‐in systems where a selective occasion is built into the process of signing the donor register, this is not the case for opt‐out systems. The loss of this selective occasion can cause significant problems where appeals to preferences motivate/legitimate shifts to opt‐out but evidence regarding variable preferences does not feed into determinations regarding organ/tissue exclusions. The nature of these problems depends on how the authorization aspect of ‘opt‐out’ systems is framed (e.g. as presumed consent, deemed consent or, given the role of familial consent in many jurisdictions as consent in name only).

## 1 INTRODUCTION

In service of aims to both increase organ supply for transplant *and* better reflect individual donation preferences,^1^ and following in the in the steps of a of a significant number of other European nations—including Austria, Belgium, Bulgaria, Croatia, the Czech Republic, Finland, France, Greece, Hungary, Italy, Latvia, Luxembourg, Norway, Poland, Portugal, the Slovak Republic, Slovenia, Spain, Sweden,^2^ and Wales—England has recently transitioned from an ‘opt‐in’ to an ‘opt‐out’ system for post‐mortem organ donation (PMOD). Consequently, while until May 2020 a prospective organ donor's willingness to donate their organs/tissues after death was previously ascertained—at least in part—by their having recorded a preference to donate on an official organ donation register prior to their deaths, it is now the case that this willingness is presumed or inferred—at least in part—from their not having recorded an objection to donation prior to their deaths on an official organ donation register.^3^

Over the last 30 years, moves from ‘opt‐in’ to ‘opt‐out’ policies have become the subject of intense debate within the philosophical and policy literature surrounding organ donation, with discussions primarily focussing on the legitimacy of such changes in the framework for authorization in organ donation,^4^ and opt‐out's potential to provide the benefits its proponents promise in terms of increased donation rates, and better reflection of donor preferences.^5^ The aim of this paper, however, is not to re‐tread old ground, but to use the recent transition to opt‐out in England to draw attention to a number of problems that have, so far, been neglected in this literature and that have as their focus the selective nature of authorization in organ donation. Therefore, using evidence from the social sciences regarding the presence and prevalence of selective preferences in PMOD, and through an exploration of how appeals to donation preferences are used to both motivate and legitimate shifts to opt‐out frameworks, as well as different ways in which the authorization aspect of opt‐out is framed, this paper draws attention to a set of problems for opt‐out organ donation frameworks that arise in contexts where:

1. Individuals demonstrate selective preferences with respect to the donation of different organs and tissues post‐mortem.
2. Legislation provides prospective donors with the opportunity to give evidence of their own selective preferences (within a ‘menu’ of options).

At this point a point of clarification is required. The fact that we are focusing on legislative contexts where an appeal to individual donor preferences is given weight should not be taken to suggest that the authorization of PMOD in these contexts is *solely* grounded in an acknowledgement of that individual's donation preferences. The preferences of parties *other* than the donor—kin and family members—are taken into account, and may in fact, have authoritative weight. However, as we explain below, the problems we expose in this paper arise where authorization takes ‘familial’ preferences into account: this is because, even in such contexts, individual preferences still have *some* weight, even if not conclusively authoritative.

The paper is structured as follows: In Section 2 we provide a short background to the paper, which surveys evidence for and research surrounding the presence and prevalence of selective preferences among prospective organ donors. This evidence is presented in order to provide useful context, and to motivate serious consideration of the problems outlined in Sections 3 and 4 regarding opt‐out's ability to take account of variable organ donation preferences. In Section 3 we explore the legitimating role that appeals to donation preferences play where the authorization aspect of opt‐out is framed in terms of presumed consent, and the extent to which selective preferences may therefore pose a problem for the legitimacy of opt‐out so characterized. In Section 4 we move on to explore the problems posed by selective preferences for opt‐out characterized in terms of ‘deemed’ consent, noting that while concerns regarding legitimacy are avoided on this characterization, concerns may still be raised regarding efficiency given that a key stated policy goal of shifts to opt‐out is that of opt‐out's potential to result in organ retrieval practices that better reflect organ donation preferences. In Section 5 we then explore how the loss of the ‘selective occasion’ poses problems for opt‐out, regardless of how its authorization aspect is framed.

## 2 SELECTIVE PREFERENCES IN PMOD: ORGAN/TISSUE SPECIFIC REFUSALS AND RESTRICTIONS

Evidence shows that preferences surrounding PMOD are more complicated than is commonly assumed (or can be adequately expressed) by reference to an individual's ‘donor’ or ‘nondonor’ status. Instead, data gathered from a number of national organ donation registers, national and regional surveys tracking PMOD willingness, and large‐ and small‐scale studies in the humanities and social sciences, increasingly indicates that willingness differs from tissue to tissue, with individuals significantly more likely to prove willing to donate certain organs and tissues for transplant post‐mortem (such as kidneys, lungs and livers) than others (such as eyes, hands and bone).

In terms of national surveys and the reports of national organ donation registers, for example, the United Kingdom's NHS Blood and Transplant ‘2020/21 Activity Report’, shows that while 85% of registered donors are willing to donate kidneys, pancreases, hearts, lungs, livers and corneas post‐mortem, 15% of this group do selectively refuse to donate at least one of these, with 10% of all donors unwilling to donate their corneas.^6^ A more detailed account of variable donation rates can also be found in a 2018 survey of knowledge attitudes and behaviour of the German population regarding organ donation, which found that of the 72% of respondents to the survey who were willing to donate organs and tissues post‐mortem 13% exhibited unwillingness to donate certain specified organs and tissues. Of this minority, rates of specific exclusion above 10% were as follows: cornea (56%) heart (27%), skin (11%).^7^ Small‐ and large‐scale studies of donation preferences in the social sciences also provide evidence of this variation with a study of US adolescents’ attitudes surrounding PMOD finding that 49% of respondents were willing to donate their organs post‐mortem but that 33% of willing donors would restrict donation and were most likely to refuse to donate the following organs and tissues: eyes (32%), pancreas (13.8%), lungs (12.8%) and heart (9.9%).^8^

Studies from the social sciences also provide information regarding variable PMOD preferences surrounding less commonly transplanted/novel/experimental organs and tissues. For example, a 2016 German study exploring students’ PMOD preferences shows that while more than 70% respondents were willing to donate their kidneys and livers, only around 30% were willing to donate a hand or a foot or a ‘large area of skin’.^9^ Similarly, a 2014 study looking to preferences and rationales for organ donation among 1,027 individuals in New Jersey shows that respondents were far more likely to prove willing to donate hearts, lungs, kidneys, corneas, than tissues such as uteri, hands and faces.^10^ A 2019 Gallup poll in the United States also points to variation in donation willingness regarding novel transplants. For, while 90.4% of respondents to the poll supported or strongly supported organ donation, only 64% and 46.9% of respondents were willing to donate their hands and faces, respectively, for transplantation post‐mortem.^11^

An additional, albeit less reliable source of evidence of selective preferences can be found in research and surveys outlining and exploring selective family refusals to donate specific organs and tissues of their relatives in countries where familial consent to donation is required prior to the retrieval of organs for transplant. For, insofar as familial refusals of organ/tissue specific consent faithfully represent individual organ donation preferences (of either the deceased or the family members themselves) and are not, for example, the result of other confounding variables—such as uncertainty regarding the wishes of the deceased, or grief and distress, and so forth—they should also be considered indicative of variable donation preferences. In the United Kingdom, for example, data collected from consent forms signed by the families of the 1,580 deceased persons who donated organs and tissue for transplant in 2019–2020 clearly shows that familial consent was far less often refused for ‘major transplantable organs’ than for tissues. Refusal rates per organ and tissue among ‘consented’ donors were as follows: kidneys (0.25%), liver (0.38%), pancreas (1.08%), lungs (2.6%), heart (3.38%), bowel (5.71%)^12^, blood vessels (5.96%), heart valves (14.66%), skin (40.34%), bone (42.99%), tendons (49.21%), corneas (51.09), meniscus (56.8).^13^ Similar data can be found in Australia and New Zealand with much lower rates of refusal for major transplantable organs—such as the liver (0%), lung (1.35%), pancreas (1.89%), heart (3.81%), and heart valve (8.55%)—than for tissues and uncommon transplants, such as the stomach and intestines (12.82%), corneas (32.17%) and bones (51.95%).^14^ Likewise, in Brazil, while no countrywide data is available, a 2020 study of authorization forms signed by the relatives of deceased organ donors between 2001 and 2016 in São Paolo shows significant variation in authorization rates for different tissues: liver (0.3%), kidneys (0.5%), heart (3.3%), lungs (5.1%), pancreas (5.5%), heart valves (14.1%), corneas (19.5%), blood vessels (46.8%), skin (57.3%) and bones (59.4%).^15^

Studies/reports of familial preferences with smaller sample sizes and/or a focus on specific organs and tissues also provide further evidence of this variation. A US study of 10,681 patient charts over a 4‐year period, for example, showed that while 46.5% of families consented to organ donation, this dropped to 34.5% for tissue donation and 23.5% for corneal donation.^16^ Canada's 2018 ‘Organ and tissue donation and transplantation system progress report’ also shows a clear difference (circa 7%) between consent rates to deceased organ donation generally (63.96%)^17^ and ‘eye and tissue donation’ specifically (57%),^18^ and a 2016 report from the United Kingdom's ocular advisory group showed eye donation rates of only 40%^19^ from deceased organ donors with family and donor refusals providing the reason for nondonation in 49.1% of cases.^20^ A 2020 study in Germany of next‐of‐kin interviews regarding organ donation showed similar consent rates to the United Kingdom (39.2%^21^), a number significantly lower than Germany's rates of familial consent recorded for organ donation generally in 2019 of 66%.^22^

No two studies, surveys or reports described above provide an identical picture with respect to the prevalence or content of selective preferences among organ donors and little to no comparative research bringing together, comparing and/or exploring general trends arising from the data is currently available. However, despite this, it is clear that a significant number of organ donors and their families *do* exhibit selectivity in their donation preferences *and* that those who do exhibit such preferences are much more likely to prove more willing to donate certain transplantable organs and tissues (such as kidneys, lungs, livers, hearts and pancreases) than others and tissues such as skin, hands, uteri, corneas, bones, and blood vessels. Research in the social sciences exploring selective refusals does go some way towards explaining this trend. In the context of corneal and eye donation, for example, interviews with donors and families who selectively refuse to donate their eyes have revealed a set of common concerns including worries about the visibility of the eyes and bodily disfigurement, their connection with identity and/or the soul, and spiritual concerns regarding the need for eyes in an afterlife.^23^ Similar concerns regarding bodily disfigurement have also been noted as underpinning refusal to donate bones and skin; a study in Israel notes that people are less willing to donate organs and tissues that are imbued with a strong connection to one's identity, or sense of self,^24^ and Williams, O'Donovan and Wilkinson additionally suggest that individuals may prove less willing to donate (or never have considered donating) organs and tissues: for nonlife saving (e.g. quality‐of‐life enhancing and/or reproductive) purposes; for relatively unknown, experimental and/or controversial transplants; and/or organs and tissues that are emotionally/socially/culturally significant.^25^

## 3 SELECTIVE PREFERENCES AND THE LEGITIMATION OF ‘OPT‐OUT’ FRAMED IN TERMS OF ‘PRESUMED CONSENT’

In the ethics and policy literature surrounding PMOD it is often claimed that opt‐out policies are liable to improve upon opt‐in policies in two key and much desired respects. First among these—given concerns regarding the global shortage of organs available for transplantation and the thousands who die every year in want of an organ transplant—are claims that opt‐out policies are likely (in many nations, at least) to provide more organs and tissues for transplant than those requiring explicit consent, moving some way towards closing the so‐called ‘transplant gap’.^26^ This potential can also be seen to constitute a key aim of the recent shift to opt‐out in England.^27^ Second, however, and more important for the purposes of this paper, are claims that opt‐out policies are likely to result in organ retrieval practices that better align with (and thus satisfy) individual preferences regarding PMOD than policies that require explicit consent for PMOD. Thought to arise due to procrastination and inertia among willing organ donors who ‘fail to get around’ to registering their preferences, such claims are supported by appeals to evidence from comparative research exploring organ donation willingness and organ donation registration rates. This research demonstrates that in nations with opt‐in PMOD policies, there is a mismatch between public sentiment and numbers of registered organ donors. Notable examples include: the United States where 90% of adults are reported to support organ donation, but only 60% are registered donors;^28^ Australia where only 34% of the population are registered donors^29^ despite ‘majority’^30^ support for the practice (circa 76% according to Transplant Australia^31^); and England where (prior to the recent policy change) despite high levels of public support for organ donation (around 80%^32^), and 65% of citizens expressing willingness regarding PMOD only 39% of citizens were registered as organ donors.^33^

Thus, in liberal societies such as our own, where it is commonly held that organ donation policy ought to track donation preferences, this data is harnessed in order to claim that the organ donation preferences of the majority will be better served by an opt‐out as opposed to an opt‐in policy. After all, if a majority of persons are in favour of organ donation, then a shift from a policy where doing nothing is either taken as indication of nothing at all *or* unwillingness to donate one's organs and tissues post‐mortem (an opt‐in policy default) to one where inaction is considered indicative of positive donation preferences (an opt‐out policy default) is likely to better reflect the donation preferences of the majority. A desire for organ retrieval policy to better reflect donation preferences is also a clearly stated motivation for the recent policy shifts to opt‐out in England. For, example, one policy document surrounding the recent change clearly states that the new opt‐out policy would ‘[change] the law in line with what the majority of people want to do’^34^and another immediately follows a description of public sentiment—‘8 out of 10 people say they would want to donate their organs and tissue after their death’—with the claim that a shift to opt‐out would ‘mean the system better reflects the position of the majority of people who would be happy to donate their organs and tissue when they die’.^35^

This potential can therefore be seen to a play a political or social legitimating role. The transition to an opt‐out framework is legitimate, and has a social license, it is suggested, because it not only (hopefully) achieves a better supply of donor organs and tissue, but it also aligns with what people *want* (though here a methodological query should be raised: should we read people's preferences from what they *say* they prefer, or from how they actually *act*?). This is certainly the case in England, where the government's response to the public consultation on implementing an opt‐out system claims that *because* ‘the vast majority of people support organ donation… it is *right* that we change the law to better reflect this’.^36^ Where the authorization aspect of opt‐out is understood in terms of presumed consent, an appeal to positive preferences surrounding PMOD not only plays the kind of motivating and social/political legitimating roles described above, but also a second related legitimating role. For it can justify the presumption of consent, or, at the very least, render such a presumption more secure.

This is the result of an important contrast between explicit and presumed consent. When someone makes a positive, explicit, act of consent there are good grounds to *believe* that their act reflects their preferences (assuming that there are no grounds to suggest coercion, manipulation, or some other restrictions on their ability to make an autonomous choice). They, after all, have taken the time and effort to record their preferences and it would be odd for a person to sign up to do something if they did not wish to do so. In contrast, when someone does not explicitly refuse their consent, we cannot so easily conclude that their inaction signals assent. For, it could also be that:

1. They would object but did not know about the nature of (or changes to) the law.
2. They would object but are ignorant of or did not properly understand what they needed to do to de‐register themselves.
3. They would object, understand the nature of the law and how to de‐register, but have simply failed to get around to registering their refusal.
4. They are ambivalent about, indifferent to, or uninterested in the choice options and thus lack a preference.

When we presume consent on the basis of an absence of refusal, we therefore make an assumption about how likely it is that inaction reflects a positive preference regarding PMOD (rather than ignorance, or inertia). For example, if we know that everybody is strongly in favour of PMOD, then the presumption that inaction reflects positive preferences is strong too (we already have the ‘major premise’ provided, as it were). However, if people's preferences are known not to be strong, when faced with individuals who have not recorded their preferences we lose our ‘major premise’. Where only a slight majority, or worse, a minority, are in favour, the fact that someone has not removed themselves from the register does not provide us with any good grounds to judge that they consent. It is as (if not more) plausible to judge that they do not know of the law, how to exercise their power to refuse, have not managed to get around to removing themselves from the register or making a decision, or that they lack preferences altogether. Evidence about favourable preferences thus matters for presumed consent by providing supporting grounds for this presumption.

Mounting evidence about variation within an individual's willingness to donate certain organs therefore seems to raise a problem for the legitimacy of a shift from explicit to presumed consent. For, if preferences for certain kinds of organ or tissue donation are weak then not only are claims regarding the potential of opt‐out to better reflect the organ donation preferences of the majority undermined, so too are claims regarding the social and political legitimacy of this shift. Variation in preferences after all, is generally downplayed or left absent from claims regarding the advantages of an opt‐out system ‘for’ donors.

As an illustration: let us imagine a country in which kidney and corneal donation policy are the province of separate legal bodies and that both are considering a shift from an opt‐in to an opt‐out policy. Let us further imagine that reliable evidence is available regarding donation preferences within the country's population with respect to PMOD generally and kidney and cornea donation specifically. This data shows overwhelming support for PMOD with 85% of citizens stating their willingness to donate organs/tissues post‐mortem, strong support for kidney donation post‐mortem (circa 75%), but that only 35% of citizens are willing to donate corneas post‐mortem. The body responsible for kidney donation could appeal to strong preferences in favour of donation generally as legitimizing a shift to an opt‐out policy and to the positive political message that opt‐out is not only liable to achieve a better supply of donor organs and tissue, but that it also and importantly aligns with the donation preferences of citizens. The body responsible for corneal donation, however, could not so readily do so. It would, after all, be an odd political justification to say: ‘Many people have strong preferences against donating their corneas, so we are switching to an opt‐out system’ and it would be misleading (if not outrightly manipulative and disingenuous) for policy makers in possession of this information to claim: ‘85% of people have strong preferences in favour of donating their organs and tissues upon death, so we are switching to an opt‐out system for cornea donation’.

Where opt‐out policies are understood in terms of presumed consent, policy makers tasked with creating and implementing opt‐out policies for organ donation *must therefore*, in order to be able to claim legitimacy (or render the presumption of consent secure), take account of selective organ donation preferences and consider the exclusion from opt‐out of organs and tissues for which low levels of donation willingness can be identified. The price of excluding such organs and tissues is clear: the retrieval of a number of transplantable organs and tissues for transplant will not be legitimate absent explicit consent from donors and/or their family members and some prospective organ/tissues recipients could potentially miss out on (or have to wait longer for) the transplantation of organs and tissues for which levels of donation willingness are low.

As discussed in Section 2, however, this is liable to prove far more challenging than in our imagined country above. For, there does not yet currently exist, in any nation, a literature that provides an accurate picture of selective preferences regarding transplantable organs and tissues. Work must therefore be undertaken to uncover and account for such preferences at the stage of policy design and, once an opt‐out policy is implemented, careful attention must be paid to any changes in public sentiment regarding organ donation over time, with adjustments to lists of organs and tissues excluded from the policy made accordingly.

Despite high administrative costs and the addition of significant complexity, the implementation of such exclusions based on evidence regarding preferences will likely not have significant detrimental effects on the availability of many organs and tissues for transplant. As can be seen in Section 2, donor willingness is high for many of the most ‘in demand’ and/or ‘vital’ organs and tissues such as kidneys, hearts, lungs and pancreases. Happily, these are therefore not strong candidates for exclusion from opt‐out policies that seek to ensure that organ retrieval practices effectively track the preferences of prospective donors. Indeed, and equally fortuitously, many of the most likely candidates for exclusion from opt‐out on such grounds are nonvital organs for which there is relatively low demand among prospective recipients, and for which longer waiting times after listing on the transplant register are possible. This, for example, is likely to be the case for reproductive tissues such as uteri, ovaries and penises; upper and lower limb and extremity transplants; and face transplants. Unfortunately, however, several routinely transplanted organs and tissues such as corneas, skin, and bones are also candidates for exclusion from opt‐out policies that restrict organs and tissues on this basis and the effects on potential transplant recipients of their exclusion may prove more serious. In the context of corneal transplantation, for example, a significant global shortage of donor corneas already exists (with only one cornea available worldwide for every 70 needed^37^) and the exclusion of corneas from opt‐out could well exacerbate this shortage.

At this point it may well be countered that policy exclusions applying at the level of organs and tissues are already in place in many nations with opt‐out organ donation policies. These include England, Scotland and Wales, which all set out long lists of organs and tissues to which opt‐out legislation does not apply, and for which explicit consent from prospective donors and/or their families post‐mortem is still required.^38^ Yet, while this is the case, concerns regarding the legitimacy of opt‐out understood in terms of ‘presumed consent’, given variable donation preferences, may only be mitigated where organ/tissue exclusions are determined by reference to such preferences. This has not been the case in England, Scotland and Wales where selected exclusions have primarily been based on considerations of ensuring legal and regulatory consistency among the constituent parts of the United Kingdom given a shared system of organ and tissue allocation; and attention directed to selective preferences has been limited to public consultation exercises with no evidence showing that account has been taken of the available social science research regarding whether and why people are (a) more willing to donate certain organs and tissues than others and (b) more willing to donate for some purposes than others.^39^

## 4 SELECTIVE PREFERENCES AND ‘OPT‐OUT’ FRAMED IN TERMS OF ‘DEEMED CONSENT

Some suggest that opt‐out frameworks for PMOD are more coherently and defensibly characterized in terms of *tacit* or *deemed*, and not presumed, consent^40^ and thus in this section we explore the problems that selective preferences may pose for opt‐out so characterized. Opt‐out characterized in terms of presumed consent rests upon the idea that consent is some kind of attitude, or state of mind.^41^ On a presumed consent framework, therefore, there is no presumption that a person has performed some kind of act aimed at permitting others to use her organs. Instead, the interpretation of inaction as signalling consent is a judgement of the form: ‘given what we know, it is reasonable to believe that this person agrees, would agree, or has a positive attitude towards, organ donation’. Where opt‐out organ donation is understood in terms of ‘deemed’ or ‘tacit’ consent, however, inaction is understood differently, not as an indicator of willingness to donate, but as an assertion of willingness, or *an act of permission*.

As Saunders, a proponent of ‘opt‐out without presumptions’, notes: consent can be given by many kinds of act, depending on context.^42^ A nod, a wink, a wave of the hand can constitute an act of consent. In some contexts, inaction—or an absence of action—can constitute consent. Saunders gives an everyday example: ‘the chairperson of a meeting may declare a motion carried if no one voices an objection, in which cases it is clear that silence implies acquiescence’.^43^ Here it is not that the chairperson *presumes* consent (based on silence), but, rather, it is that in that context, the chair is specifying the kind of act that constitutes consent. Saunders then argues:

It is up to an appropriate authority to determine what counts as consent in a given context. In a board meeting the chair (or constitutional rules) may specify that consent is shown by the raising of one's hand, saying ‘aye’, or even silence. Similarly, assuming that the state is a legitimate authority, then it is up to the state to specify how consent can be shown. Different states legitimately have different procedures, concerning donor registers, family vetoes and so on. If the state declares that not opting out of an organ donation scheme will be interpreted as consent, then those who do not opt out implicitly consent.^44^

Den Hartogh makes a similar point when discussing opt‐out organ donation in a 2019 paper, noting that it is possible

to interpret ‘deeming’ to have a declaratory rather than an epistemic meaning: the law declares that a person who does not register an objection thereby authorises the removal of his organs. And if the person knows his registration and its legal meaning, that it not a legal fiction. In many other contexts inside and outside of the law, tacit consent is one valid form of consent. What counts as valid consent is, within some general constraints, largely a matter of convention or law.^45^

Framing opt‐out organ donation in terms of tacit consent, or interpreting deemed consent as having a declaratory meaning, seems to avoid the problems with presumed consent introduced in Section 3. To restate: on a presumed consent framework, the interpretation of inaction as consent is a judgement of the form: ‘given what we know, it is reasonable to believe that this person agrees with, or has a positive attitude towards organ donation’. As we saw above, knowledge of preferences matters as part of the legitimation of such a judgement. In contrast, if a person's inaction *constitutes* consent, as it does when the authorization aspect of opt‐out is understood in terms of ‘tacit’ or ‘deemed’ consent, then, as Saunders and Den Hartogh suggest, their actual or likely attitudes and preferences are not directly relevant to answering the question ‘Has this person consented?’. The answer to that question has already been fixed: inaction constitutes consent, and what one's attitudes, intentions or preferences are, has nothing to do with it as ‘consent is not about subjective intentions’.^46^ Because on this view an individual's subjective preferences and intentions regarding organ donation generally are irrelevant to determinations of consent and refusal, we might assume that similar conclusions should be drawn with respect to preferences and intentions regarding the donation of specific organs and tissues. Unlike where opt‐out is characterized in terms of presumed consent, where evidence about weak or negative preferences undermines the presumption of consent, if an opt‐out framework is framed in terms of *deemed or tacit* consent then the existence of variation in preferences need not be considered to pose a problem.

The legitimacy of an opt‐out policy for organ donation framed in terms of deemed consent may therefore not so strongly rest on its ability to reflect donor preferences in the same way that is required on a ‘presumed consent’ framework. However, despite this it may still be the case that failure to reflect organ donation preferences when determining the scope of organ donation policy is problematic on other grounds and that similar prescriptions should be made regarding policy exclusions regardless of whether opt‐out is framed in terms of ‘presumed’, ‘deemed’ or ‘tacit’ consent. One such ground is highly likely to regard efficiency: the extent to which the proposed version of an opt‐out policy (including scope constraints such as organ and tissue exclusions) are likely to align with and thus forward (or disrupt and thus threaten) the stated rationales and aims underpinning the policy shift. As explained in Section 3, the major stated policy goals of shifts from opt‐in to opt‐out policies tend to be twofold in nature: to both increase the supply of organs and tissues for transplant *and* to achieve donation outcomes that better reflect the preferences of prospective organ donors regarding PMOD than ‘opt‐in’ systems. This, as discussed above, was certainly the case in policy documents surrounding the recent English transition. Thus, decisions regarding scope constraints in countries where opt‐out has been implemented in service of these aims should, ceteris paribus, also be informed by these aims. Another concern has transparency and the maintenance of public trust in organ donation as its focus. For, where positive preferences regarding PMOD are clearly appealed to as motivating a proposed policy shift, to then wilfully ignore evidence regarding selective donation preferences at the design stage, seems to point to alternative policy goals and thus to political spin and disingenuity, which may ultimately (and seriously) threaten public trust in organ donation.

## 5 OPT‐IN, OPT‐OUT AND THE DISAPPEARANCE OF A SELECTIVE OCCASION

As seen in Sections 3 and 4, authorization processes for PMOD are often framed in terms of consent, with opt‐in systems for organ donation classified as based on ‘express’/‘explicit’ consent, and opt‐out systems for organ donation classified as based on ‘presumed’, ‘tacit’ or ‘deemed’ consent. On these views, the authorization aspect of organ donation might be considered indistinguishable from other kinds of consent, such as sexual consent and consent to medical treatment. In such contexts adults with capacity are considered to have a sole and authoritative power to consent to (or refuse) the act or intervention in question and their individual act of consent (or refusal) constitutes ‘the last word’ regarding its permissibility. This, however, is often not the case in the context of PMOD. For, while it is possible for consent to PMOD to be sole and authoritative (such as in a ‘mandated choice’ system or a ‘hard’ ‘opt‐in’ or ‘opt‐out’ system where individual preferences are decisive, and any conflicting preferences of the deceased's relatives are not taken into account), in practice familial preferences are given significant—and often overriding—weight at the point of donation. Thus, it may be considered that acts of authorization for PMOD are more appropriately viewed as constituting, what we term, ‘consent in name only’. Such acts of authorization are *indicative* and *evidential* of a preference to donate, and play an important epistemic role, but are neither sole nor authoritative, having only a limited normative significance through feeding into later and decisive deliberations among relatives (or other legally determined proxies) and medical professionals that will ultimately determine permissibility at the point of donation.

In England, for example, the Human Tissue Act as amended by the Organ Donation (Deemed Consent) Act 2019, considers ‘appropriate consent’ to donation to be present where: (a) prospective donors have recorded positive donation preferences prior to their death (e.g. signed the organ donor register), or (b) have not registered an objection to donation prior to their death^47^ absent compelling information to the contrary from a person who stands in a qualifying relationship to the deceased.^48^ However, this is *not* the case in practice and a far greater role is provided to relatives than legislation provides. For, as explained in the HTA code of practice, ‘The existence of appropriate consent permits donation to take place, but does not mandate that it must. The final decision about whether to proceed rests with… the medical practitioners caring for the patient, in conversation with the family’.^49^ Thus, it is explained that while ‘individuals have the autonomous right to give or refuse consent to all or any of their organs or tissue being used for transplantation after their death’,^50^ and ‘the family cannot revoke legally valid consent’,^51^ the views and preferences of the family ‘will always be taken into account throughout the donation process and will have a strong influence on whether or not donation proceeds’.^52^ This is echoed by NHS Blood and Transplant who state that while family members ‘do not have the legal right to veto or overrule’^53^ a relative's organ donation decisions ‘There may, nevertheless, be cases where it would be inappropriate for donation to go ahead if donation would cause distress to your family’.^54^

Given worries about framing the authorization aspect of ‘opt‐out’ organ donation policies in terms of ‘consent’, we now wish to introduce a problem for opt‐out posed by the existence of variable donation preferences that arises *regardless of framing* (e.g. as deemed consent, presumed consent, or as above, as ‘consent in name only’), and does not, importantly, similarly arise on opt‐in frameworks. This concerns the fact that while an explicitly selective occasion is generally built into the process of signing the donor register on opt‐in systems for PMOD, this is not the case on opt‐out systems. On opt‐out systems the state determines the scope of one's consent unless one chooses to explicitly opt‐out of the donation of all/particular organs and tissues. This, we suggest, is a problematic loss, as selective occasions of this kind play at least four important roles:

1. They direct a prospective organ donor's attention to the *existence* of the option to shape their donation wishes, to the fact that donation is not an ‘all or nothing’ affair.
2. They direct the prospective donor's attention to *which* options are available, by specifying which types of organ and tissue *may* be selected.
3. They provide the prospective donor with the opportunity to consider their donation preferences and deliberate (albeit minimally) as part of the process of recording their preferences.
4. They provide the prospective donor with the opportunity to indicate and make explicit their specific, selective, donation wishes, which can then feed into the deliberations of families and donor physicians.

Let us be a little clearer about what this ‘selective occasion’ is, and why it matters. On both opt‐in and opt‐out systems there is what we might think of as a ‘path’ to authorization. The path is a complex, social (and institutional) one. On an opt‐in system, the provision of a selective occasion is ‘built in’ to the path to authorization and a prospective donor's preferences are recorded only after an individual is put into a position to attend to and reflect upon their options.^55^

First, it is important to note that the selective occasion can be structured in different ways. For example, one kind of selective occasion is where the default is ‘donate all’ with then some further options to find out more, and select (by opening new boxes, windows, links). A different kind of selective occasion is where the donor has to select (or ‘tick’) *each* category that they are willing to donate. Here the selective nature of the occasion is very tightly ‘built in’ to the process: if the donor is capable of reading, then, albeit minimally, they *cannot help* but be aware of the fact that they are indicating a willingness to donate kidneys *and* corneas, say. The evidential value of a selective occasion clearly depends upon the facts about how the selective occasion is constructed. Second, for any particular configuration of a selective occasion, different individuals will *engage* with, or respond to, the occasion in different ways (though, in light of the point just above, the ‘leeway’ for variation in response will depend upon how things are structured in offering the selective options). A diligent, worried, person might behave in one way, carefully considering the options, and seeking more information to make an informed decision. Another donor might simply ‘click through’ the options without much reflection or deliberation at all.

Although these points of variation should be noted, it is arguable that there is still a *fundamental* contrast between opt‐in and opt‐out frameworks. Opt‐in frameworks at the very least *provide* some kind of deliberative occasion, which plays the role of directing attention, making options known, and allowing an indication of selective preferences to be given *as part of* the complex social path to authorization. For any particular individual there may be an open question about how much they have engaged with such a process, but *in general*, the provision of such occasions affords better grounds for making inferences about a donor's actual selective preferences, better, that is, than the evidence that is provided on opt‐out.

The social path to authorization on opt‐out is one that has a certain kind of *inaction* playing the role of the positive act of registration that we find in opt‐in. Individual donors may, of course, make an informed decision that their inaction reflects their selective preferences (to donate all available types of organs and tissues), but there is nothing analogous to the provision of the selective occasion *built into* the path to authorization. It is not as if the inaction of the donor on an opt‐out framework is triggered by, or bundled with, some kind of attention‐directing, information‐giving, preference‐signalling *occasion*. The point, then, is that there is a contrast between the two broad *types* of path to authorization, where one includes a selective occasion as part of the path, the other does not. This would be of no significance at all if it were the case, say, that *only* opt‐in frameworks were selective (whilst all opt‐out frameworks were simply an ‘all or nothing’ affair). But this is not the case. Opt‐out frameworks are selective but fail to provide as strong evidence of *actual* selective preferences as opt‐in frameworks and this is the case whether or not things are framed in terms of consent, and independent of the power of family veto.

To illustrate, let us compare the new ‘opt‐out’ framework for PMOD in England with the previous ‘opt‐in’ framework for PMOD, a framework that was (or aimed to be) explicitly selective. On the previous opt‐in framework if an individual sought to register their donation wish online, the first thing they encountered after providing some personal details was the question: ‘Do you want to donate all of your organs and tissue?’ At the side, an explanatory note stated: ‘You can select to donate some, or all of your organs and tissue’. The registrant was then provided with two choice options: ‘all’ or ‘some’. Where she chose ‘all’ she was informed that: ‘By selecting “all” you may be able to help up to nine people through organ donation, and even more through tissue donation. Thank you’. Where the registrant chose ‘some’, however, she was given a list of eight organ and tissue ‘labels’ (kidney, corneas, heart, lungs, liver, pancreas, small bowel, and tissue), with further options to learn more about what those ‘labels’ referred to before recording her choice. This process provided the interested party with an explicitly selective occasion (albeit one that was suboptimal given that the only way to discover what ‘all’ or ‘some’ included was to select ‘some’ as this was the only way to make the list of organ and tissue ‘labels’ visible).^56^ Thus, on this system, where a person did authorize donation, permission was provided only *after* they were put into a position to reflect on their preferences and exercise control. On the opt‐in framework, authorization follows a selective occasion, and its content and scope reflect—or, at least ought to reflect—the results of selective attention and deliberation, however minimal, about preferences. On the new opt‐out system, however, while it is possible to both opt‐in to, and opt‐out of donation, not only are we likely to lose the explicit act by which a general willingness to donate one's organs/tissues after death is made manifest, we are also likely to lose the selective occasion.

It is arguable that we are apt to be misled here if we frame things in terms of a contrast between different kinds of *consent*. That is, suppose we think of the contrast between opt‐in and opt‐out in terms of being a contrast between, on the one hand ‘explicit’ or ‘express’ consent, and, on the other ‘presumed’ or ‘deemed’ consent (acknowledging that there are differences between the latter two). Conceived of thus, it may seem that we have two different paths to authorization, which are routed via different kinds of action (or inaction). But this is to focus on ‘consent’ as the authorizing element, rather than to view the path to authorization as a more complex social process. The more complex social process is one where *evidence* of preferences is fed into consultations with family post‐mortem. Although such evidence is never conclusive, it is *stronger* evidence that someone wants to donate their corneas if they have been presented with a well‐structured selective occasion where their attention has been drawn to that option (and, say, where the default is *not* to donate) than the strength of evidence that one has from a person's inaction (where no selective occasion has been a causal part of the provenance of, or context of, that inaction).

Insofar as opt‐out systems encourage donors to discuss their preferences with their families it may be that information about preferences is ‘fed through’ to the ultimate decision‐making context via a range of communicative routes *within* the family. However, insofar as organ donation is framed in terms of an ‘all or nothing’ affair, without attention being drawn to the selective options that are *in fact* available, such a diffuse, informal network of communication is unlikely to provide as strong evidence of individual selective preferences as that which is provided when it is known that the (potential) donor has been exposed to a selective occasion where such options have been made known (and, perhaps, where the expression of preferences has to ‘feed through’ an explicit process of at least minimal deliberation, at least to the extent ‘Should I tick this box that says “corneas” or not?’).

## 6 CONCLUSIONS

Within this paper we have sought to draw attention to a set of previously unexplored problems for opt‐out organ donation frameworks that arise in contexts where:

1. Individuals demonstrate selective preferences with respect to the donation of different organs and tissues post‐mortem.
2. Legislation provides prospective donors with the opportunity to give evidence of their own selective preferences (within a ‘menu’ of options).

Where opt‐out is understood in terms of presumed consent, the existence of variable preferences surrounding PMOD has the potential to cast significant doubt on the legitimacy of a policy shift from opt‐in to opt‐out. For, evidence about favourable donation preferences matters for presumed consent by providing supporting grounds for this presumption and where preferences in favour of the donation of specific organs and tissues are weak then, so too are claims regarding the social and political legitimacy of this shift. Thus, to claim legitimacy, policy makers must take account of selective organ donation preferences and ensure that organs and tissues for which low levels of donation willingness can be identified are excluded from opt‐out policies.

For those who believe opt‐out is more defensibly characterized in terms of ‘deemed’ or ‘tacit’ consent’, variable preferences surrounding PMOD do not pose the same problems of legitimacy. For, on a deemed/tacit consent framework inaction constitutes consent, and what one's attitudes, intentions or preferences are has nothing to do with it. However, given the stated goals underpinning shifts to opt‐out organ donation policies in many nations (including England), a failure to account for selective preferences when determining the content of opt‐out policy would likely significantly and negatively impact policy efficiency and could also threaten public trust in organ donation. Whether or not a failure to account for selective preferences in opt‐out policies will undermine public trust in organ donation is, of course, an empirical question, which can only be answered with the passage of time.^57^

As a result, prudence requires policy makers to ensure (as where opt‐out is characterized in terms of presumed consent) that decisions regarding the kinds of organs and tissues included within/excluded from opt‐out policies are based on reliable data regarding the presence and prevalence of selective preferences among citizens. As discussed in Section 2, however, taking account of selective preferences when designing opt‐out policies is liable to prove far more challenging than might be assumed. For, there does not currently exist, in any nation, a literature that provides an accurate picture of selective preferences regarding transplantable organs and tissues and as a result, selective preferences are liable to cause significant administrative and organizational challenges that cannot be addressed until such time that this information becomes readily available.

While the aforementioned problems may be reduced over time, through concerted efforts to both uncover and account for selective preferences at the policy design stage, the existence of variable preferences regarding PMOD also poses an additional, and less readily resolved problem for opt‐out organ donation. This applies regardless of how we characterize the authorization aspect of opt‐out: in terms of ‘presumed’ consent, ‘deemed’ consent, or ‘consent in name only’ and has as its focus the selective occasion that is ‘built in’ to the path to authorization on opt‐in systems for PMOD, but that is not built into the path to authorization on opt‐out. Given the epistemically significant role of this occasion—which directs a prospective organ donor's attention to the existence of the option to shape their donation wishes, provides them with the opportunity to consider their donation preferences and make explicit their donation wishes, and provides valuable evidence to families and donor physicians who may have a significant role in final decisions regarding organ donation—this loss is to be regretted by all who consider that respect for individual donation preferences should drive organ donation policy.

Finally, in this paper we have sought to introduce a problem (or potential problem) for further discussion, one that seems not to have been noticed in the voluminous debates about the proper framework for PMOD. There are, of course, further questions raised about the seriousness of this problem (or problems) and about what the proper and appropriate response to such problems ought to be. We have not space to address these here but, in line with our discussion above, we note that an adequate discussion will require more wide‐ranging evidence, not just about what donor preferences are, but about the strength and significance of such preferences *alongside* other relevant preferences (after all, in response to a questionnaire about donor preferences one may indicate a preference that one not donate one's corneas, but *in the round*, such a first‐order preference might be readily be out‐weighed by a willingness to ‘do the right thing’). Whatever direction future research may take, we hope that the discussion here at least motivates more research in this area and gives an initial prompt to further debate.

## CONFLICT OF INTEREST

The authors declare no conflict of interest.

## Footnotes

1 See, for example: Department of Health and Social Care. (2018a). *The new approach to organ and tissue donation in England: Government response to public consultation*. Department of Health and Social Care. https://assets.publishing.service.gov.uk/government/uploads/system/uploads/attachment_data/file/731913/govt-response-organ-donation-consent.pdf; Department of Health and Social Care. (2018b). *Consultation outcome: Consultation on introducing ‘opt‐out’ consent for organ and tissue donation in England*. https://www.gov.uk/government/consultations/introducing-opt-out-consent-for-organ-and-tissue-donation-in-england/consultation-on-introducing-opt-out-consent-for-organ-and-tissue-donation-in-england

2 Rithalia, A., McDaid, C., Suekarran, S., Norman, G., Myers, L., & Sowden, A. (2009). A systematic review of presumed consent systems for deceased organ donation. *Health Technology Assessment, 13*(26), 47–53.

3 Organ Donation (Deemed Consent) Act, 2019.

4 For a clear summary of debates in this arena see: Wilkinson, T. M. (2011). *Ethics and the acquisition of organs*. Clarendon Press, pp. 81–101.

5 See, for example: Thaler, R. H., & Sunstein, C. R. (2009). *Nudge: Improving decisions about health, wealth, and happiness*. Penguin, pp. 184–192.

6 NHS Blood and Transplant. (2021). *Organ donation and transplantation activity report 2020/21*. NHS Blood and Transplant, p. 133. https://nhsbtdbe.blob.core.windows.net/umbraco-assets-corp/19481/activity-report-2019-2020.pdf

7 Caille‐Brillet, A. L., Zimmerling, R., & Thais, H. M. (2019). *Wissen, Einstellung und verhalten der allgemeinbevölkerung zur organ‐ und gewebespende: Ergebnisse der Repräsentativbefragung 2018*. Bundeszentrale für gesundheitliche Aufklärung (BZgA), pp. 23, 32–33.

8 Baughn, D., Rodrigue, J. R., & Cornell, D. L. (2006). Intention to register as organ donors: A survey of adolescents. *Progress in Transplantation, 16*(3), 264.

9 Wöhlke, S., Inthorn, J., & Schicktanz, S. (2016). The role of body concepts for donation willingness. insights from a survey with German medical and economics students. In R. J. Jox, G. Assadi, & G. Marckmann (Eds.), *Organ transplantation in times of donor shortage – Challenges and solutions* (pp. 27–49). Springer, p. 36.

10 Sarwer, D. B., Ritter, S., Reiser, K., Spitzer, J. C., Baumann, B. M., Patel, S. N., Mazzarelli, A. J., Levin, S., Doll, S., & Caplan, A. L. (2014). Attitudes toward vascularised composite allotransplantation of the hands and face in an urban population. *Vascularised Composite Allotransplantation*, *1*(1–2), 26.

11 Department of Health and Human Services. (2020). *2019 National survey of organ donation attitudes and practices: Report of findings*. U.S. Department of Health and Human Services, Health Resources and Services Administration, pp. 21, 51. https://www.organdonor.gov/sites/default/files/organ-donor/professional/grants-research/nsodap-organ-donation-survey-2019.pdf

12 NHS Blood and Transplant. (2020). *Organ donation and transplantation activity report 2019/20*. NHS Blood and Transplant, pp. 26–28. https://nhsbtdbe.blob.core.windows.net/umbraco-assets-corp/19481/activity-report-2019-2020.pdf

13 A. Newton (NHS Blood and Transplant Statistical Enquiries Service, personal communication, November 26, 2020).

14 Excell, L., Marion, V., & Russ, G. (Eds.). (2012). *Australia and New Zealand organ donation registry (ANZOD registry) 2012 report*. Australia and New Zealand Organ Donation Registry. https://www.anzdata.org.au/report/anzod-annual-report-2012/. NB: Data from the 2012 ANZOD Registry Report is used here as since 2013 ANZOD reports have not contained the data regarding bone, tissue and corneal donation consent rates relevant to this publication.

15 Dos Santos, M. J., Leal de Moraes, E., Santini Martins, M., Carlos de Almeida, E., Borges de Barros E. Silva, L., Urias, V., Silvano Corrêa Pacheco Furtado, M. C., Brito Nunes, Á., & El Hage, S. (2018). Trend analysis of organ and tissue donation for transplantation. *Transplantation Proceedings, 50*(2), 392.

16 Siminoff, L. A., Arnold, R. M., & Hewlett, J. (2001). The process of organ donation and its effect on consent. *Clinical Transplantation, 15*(1), 39–47.

17 Canadian Blood Services. (2018). *Organ and tissue donation and transplantation system progress report 2018*. Canadian Blood Services, p. 11. https://profedu.blood.ca/sites/msi/files/organ_and_tissue_donation_and_transplantation_-_system_progress_report_2018.pdf

18 Ibid: 20.

19 NHS Blood and Transplant Ocular Tissue Advisory Group. (2016). *Eye procurement from solid organ donors activity report 2016*. NHS Blood and Transplant, p. 1. https://nhsbtdbe.blob.core.windows.net/umbraco-assets-corp/2790/eye_procurement_solid_organ_donors_june16.pdf

20 Ibid: 5.

21 Schmidt, S., Ghouzi, A., Petrov, G., Hofmann, N., Boergel, M., & Sixt, S. U. (2020). Cornea donation in Germany: Obtaining consent. *Clinical Transplantation, 34*(8), 1.

22 Deutsche Stiftung Organtransplantation. (2019). *Organspende und Transplantation in Deutschland 2019*. Deutsche Stiftung Organtransplantation, p. 33. https://www.dso.de/SiteCollectionDocuments/DSO-Jahresbericht%202019.pdf

23 Lawlor, M., Kerridge, I., Ankeny, R., Dobbins, T. A., & Billson, F. (2010). Specific unwillingness to donate eyes: The impact of disfigurement, knowledge, and procurement on corneal donation. *American Journal of Transplantation, 10*(3), 657–633.

24 Sperling, D., & Gurman, G. M. (2012). Factors encouraging and inhibiting organ donation in Israel: The public view and the contribution of legislation and public policy. *Journal of Bioethical Inquiry, 9*(4), 479–497.

25 Williams, N. J., O'Donovan, L., & Wilkinson, S. (2022). Presumed dissent. Opt‐out organ donation and the exclusion of organs and tissues. *Medical Law Review*, 11–12. https://academic.oup.com/medlaw/advance-article/doi/10.1093/medlaw/fwac001/6529455?login%3Dtrue

26 In *Nudge*, for example, Thaler and Sunstein claim that that switching from explicit consent to opt‐out ‘would save thousands of lives every year’. Thaler & Sunstein, op. cit. note 5, p. 189.

27 See: for example: Department of Health and Social Care (2018a), op. cit. note 1, p. 6; Department of Health and Social Care. (2019). *Organs and tissues to be excluded from the new system of organ and tissue donation in England (known as “opt‐out” or “deemed” consent)*. Department of Health and Social Care, p. 8. https://assets.publishing.service.gov.uk/government/uploads/system/uploads/attachment_data/file/798467/Organs_and_tissues_to_be_excluded_from_the_new_system_of_organ_and_tissue_donation_in_England_-_consultation_document.pdf; Department of Health and Social Care. (2018). *Impact assessment: An opt‐out system of organ and tissue donation*. Department of Health. https://assets.publishing.service.gov.uk/government/uploads/system/uploads/attachment_data/file/731915/Organ_donation_impact_assessment.pdf

28 Health Resources and Services Administration. (2021). *Organ donation statistics*. https://www.organdonor.gov/statistics-stories/statistics.html

29 Australian Government Organ and Tissue Authority. (2020). *2020 Australian donation and transplantation activity report*. Australian Government Organ and Tissue Authority, p. 9. https://www.donatelife.gov.au/sites/default/files/2020_australian_donation_and_transplantation_activity_report.pdf

30 Ibid.

31 Transplant Australia. (2019). *The facts*. https://transplant.org.au/the-facts/

32 Department of Health and Social Care (2018a), op. cit. note 1, p. 6.

33 NHS Blood and Transplant, op. cit. note 12, p. 128.

34 Department of Health and Social Care (2019), op. cit. note 27, p. 8.

35 Department of Health and Social Care (2018b), op. cit. note 1.

36 Department of Health and Social Care (2018a), op. cit. note 1, p. 7.

37 Gain, P., Jullienne, R., He, Z., Aldossary, M., Acquart, S., Cognasse, F., & Thuret, G. (2016). Global survey of corneal transplantation and eye banking. *JAMA Ophthalmology, 134*(2), 167.

38 See, for England, Scotland and Wales: (Permitted Material: Exceptions) (England) Regulations 2020; Human Transplantation (Excluded Relevant Material) Regulations 2015; Human Tissue (Excepted Body Parts) (Scotland) Regulations 2020.

39 See, for example: Department of Health and Social Care (2019), op. cit. note 27, Department of Health and Social Care. (2020)*. Government response to the consultation on the draft human tissue (Permitted Material: Exceptions) (England) Regulations 2020*. Department of Health and Social Care. https://assets.publishing.service.gov.uk/government/uploads/system/uploads/attachment_data/file/867918/Government_response_to_opt-out_organ_donation_-_organs_and_tissues_excluded_from_the_new_system.pdf; Scottish Government. (2020a). *Human tissue (Authorisation) (Excepted Body Parts) (Scotland) Regulations 2020: Consultation*. Scottish Government. https://www.gov.scot/publications/consultation-human-tissue-authorisation-excepted-body-parts-scotland-regulations-2020/documents/; Scottish Government. (2020b). *Human tissue (Authorisation) (Excepted Body Parts) (Scotland) Regulations 2020: Consultation analysis*. Scottish Government. https://www.gov.scot/publications/human-tissue-authorisation-excepted-body-parts-scotland-regulations-analysis-consultation-responses/; Welsh Government. (2020). *Human transplantation (Excluded Relevant Material) (Wales) (Amendment) Regulations: Consultation on draft regulations*. Welsh Government. https://gov.wales/sites/default/files/consultations/2020-02/consultation-document_0.pdf; Welsh Government. (2021). *Human transplantation (Excluded Relevant Material) (Wales) (Amendment) Regulations: Consultation Summary Report*. Welsh Government. https://gov.wales/human-transplantation-excluded-relevant-material-wales-amendment-regulations-consultation-summary; Williams et al., op. cit. note 25, pp. 1–31.

40 See, for example: Den Hartogh, G. (2011). Can consent be presumed? *Journal of Applied Philosophy, 28*(3), 295–307; Den Hartogh, G. (2019). Respect for autonomy in systems of post‐mortem organ procurement: A comment. *Bioethics, 33*(5), 550–556.

41 Den Hartogh (2019), op. cit. note 40, p. 552.

42 Saunders, B. (2012). Opt‐out organ donation without presumptions. *Journal of Medical Ethics, 38*(2), 69–72.

43 Ibid: 71.

44 Ibid: 71.

45 Den Hartogh (2019), op. cit. note 40, p. 556.

46 Saunders, op. cit. note 42, p. 71.

47 Human Tissue Act (2004) As amended by the Organ Donation (Deemed Consent) Act, 2019. s. 3.

48 Ibid: s. 3.6B.

49 Human Tissue Authority. (2021). *Codes of Practice and Standards. Code F Donation of solid organs and tissue for transplantation. Part two – deceased organ and tissue donation*. Human Tissue Authority (para 118). https://content.hta.gov.uk/sites/default/files/2020-11/Code%20F%20part%202.pdf

50 Ibid: para 42.

51 Ibid: para 89.

52 Ibid.

53 NHS Blood and Transplant. (2021). *Understanding consent for organ donation*. https://www.organdonation.nhs.uk/helping-you-to-decide/about-organ-donation/consent/

54 Ibid.

55 The provision of a selective occasion does not, of course, imply that all donors will, in fact, fully attend to, reflect upon or deliberate upon, such options.

56 NHS Blood and Transplant. (2019). *Register your details – Yes I donate*. https://web.archive.org/web/20190426234046/, https://www.organdonation.nhs.uk/register-your-decision/register-your-details/

57 Despite this, precedent does suggest that a mismatch between the expectations of donors and/or their families, and organ donation policy can lead to public scandal and associated reductions in donation rates. One key example of this are the clear reductions in public trust and organ donation rates that were observed in France after the so‐called ‘Amiens Affair’ in the early 1990s. The case centred around a legal complaint lodged by the parents of 19‐year‐old Christophe Tesniere, after the legally permitted (at the time) but not explicitly parentally sanctioned removal of their son's eyes for PMOD and is thought to have contributed significantly to a 38% fall in corneal donation rates (from 3,774 in 1991 to 2,383 in 1993). See, for more information: Patel, T. (1999, July 2). France's troubled transplant trade. *New Scientist*.https://www.newscientist.com/article/mg13918802-700/; Tuppin, P., Boutin, D., Vienne, P., Auvert, B., & Loty, B. (1997). National census of corneal donations, waiting lists, and transplantation in France. *Transplant Proceedings, 29*(1–2), 996–997; Tuppin, P., & Loty, B. (1999). The progress of cornea donation and transplantation in France. *Cornea, 18*(6), 682–685.

